# Differential Modulation of the Ileal Bile Acid Transporter ASBT by Fatty Acids

**DOI:** 10.3390/ijms27219920

**Published:** 2026-11-05

**Authors:** Pooja Malhotra, Zeba Farooqui, Alexander Ticho, Shane Comiskey, Aisha Qazi, Nathan Calzadilla, Ricardo J. Suarez, Ravinder K. Gill, Waddah A. Alrefai

**Affiliations:** 1 Division of Gastroenterology & Hepatology, University of Illinois Chicago, Chicago, IL 60612, USAzeba25@uic.edu (Z.F.);; 2 Division of Gastroenterology & Hepatology, Mayo Clinic, Rochester, MN 55905, USA; 3 Jesse Brown VA Medical Center, Chicago, IL 60612, USA

**Keywords:** ASBT, intestinal DHHC acyltransferases, omega-3 fatty acids, bile acid absorption

## Abstract

The ileal ASBT bile acid transporter is a critical mediator of bile acid and cholesterol homeostasis and is modulated by fatty acids via S-acylation. We investigated mechanisms underlying differential regulation of ASBT by saturated and polyunsaturated fatty acids. Using a prenylation mimetic with a CAAX box at the C-terminus of ASBT, we showed that transport activity in transfected HEK293 cells increased significantly, suggesting that direct attachment of a saturated fatty acid stimulates ASBT function. Furthermore, siRNA-mediated attenuation of DHHC21 acyltransferase expression reduced ASBT S-acylation and its function, demonstrating DHHC21 as a positive regulator of ASBT function. Conversely, treatment with omega-3 polyunsaturated fatty acids (n-3 PUFAs), like eicosapentaenoic acid (EPA), reduced ASBT function in HEK293 cells. Supplementation of fish oil, as a source of n-3 PUFAs, to a high-fat, high-cholesterol diet fed to C57BL6/J mice protected them against diet-induced hepatic steatosis, along with reduced bile acid absorption consistent with the inhibition of ASBT. This was supported by increased fecal bile acids, decreased ileal bile acid-stimulated gene expression, and increased hepatic bile acid synthesis-related gene expression. Metabolic labeling with alkyne-EPA in HEK293 cells showed, for the first time, direct binding of this n-3 PUFA to ASBT. Unlike palmitate modification, EPA binding was highly resistant to hydroxylamine retrieval, indicating a mechanism distinct from S-acylation. Our data unraveled a novel role for DHHC21 in regulating ASBT and provided evidence for distinct mechanisms by which saturated palmitate and polyunsaturated EPA fatty acids bind ASBT. The direct effect on ASBT and inhibition of bile acid absorption may partly contribute to the protective effects of n-3 PUFAs on the liver.

## 1. Introduction

The ileal apical sodium-dependent bile acid transporter ASBT mediates the rate-limiting step in bile acid reabsorption in the ileum to maintain their cycling between the small intestine and liver [1,2,3]. Bile acids are critical for emulsifying lipids and facilitating their absorption [1]. A recent study showed that individual bile acid species exert differential effects on fatty acid absorption, and that a reduction in the total bile acid pool decreases the absorption of saturated, but not unsaturated, fatty acids [4]. Notably, lower ASBT function markedly reduces bile acid pool size and alters bile acid composition [5]. Therefore, altering ASBT may also lead to differential effects on fatty acid absorption [6]. In light of potential bidirectional effects, it is reasonable to postulate that fatty acids have differential effects on bile acid absorption.

Given the central role of ASBT in maintaining bile acid homeostasis and influencing lipid absorption, ASBT has been extensively investigated as a therapeutic target for managing metabolic and lipid- and cholesterol-related disorders [1,2]. Previous studies have shown that Inhibiting ASBT lowers plasma cholesterol [7], improves glycemic control in type 2 diabetes mellitus [8], and protects against diet-induced metabolic dysfunction-associated steatotic liver disease (MASLD) [9]. However, these results were variable [10,11,12], suggesting the involvement of yet-uncharacterized regulatory factors that may influence the response to ASBT inhibition. Indeed, recent studies in mouse models showed that the efficacy of ASBT inhibitors in preventing MASLD varied depending on the type of fatty acids enriched in the diet. For example, ASBT-mediated effects on metabolic dysfunction were greater with a diet rich in saturated fatty acids than with a diet rich in polyunsaturated fatty acids [13]. Taken together, these prior findings underscore the critical role of crosstalk between fatty acids and ASBT in shaping bile acid homeostasis and dictating the response to ASBT inhibitors in diseases such as MASLD and diabetes mellitus.

Regarding potential mechanisms, previous studies have demonstrated a post-translational effect of fatty acids on ASBT via S-acylation [14,15]. This reversible modification involves the direct binding of fatty acids to cysteine residues in the polypeptide via thioester bonds, altering protein function, conformation, and localization within specific plasma membrane domains [16]. ASBT was shown to be S-acylated at basal levels across different cell lines and in native mouse and human intestine, and ASBT activity was reduced by an S-acylation inhibitor [14,15]. A family of acyltransferases that catalyze protein S-acylation has been identified, comprising 23 isoenzymes (DHHC1-24) that contain a conserved Asp-His-His-Cys (DHHC) domain and exhibit unique tissue- and subcellular-specific distributions [16]. Although S-acylation appears critical for ASBT functioning [14,15], the DHHC enzyme(s) involved remain unknown. ASBT activity was shown to be enhanced by saturated (e.g., palmitate) and reduced by monounsaturated (e.g., oleate) fatty acids [14]. However, the effects of other fatty acids, including omega-3 polyunsaturated fatty acids (n-3 PUFAs), on ASBT function remain elusive.

Our current studies have shown, for the first time, the unique differential expression of the DHHC acyltransferases across all segments of the GI tract and provided novel evidence for the role of DHHC21 in ASBT S-acylation. We further demonstrated that n-3 PUFAs inhibited ASBT function and bile acid absorption in both in vitro and in vivo mouse models via direct binding to ASBT independent of S-acylation. Our findings offer novel insights into regulatory factors that could serve as adjunct therapeutic targets to enhance the effectiveness of ASBT inhibition in metabolic disorders and MASLD.

## 2. Results

### 2.1. ASBT Prenylation Mimetic Elicits Increased Transport Activity

**Figure 1:**
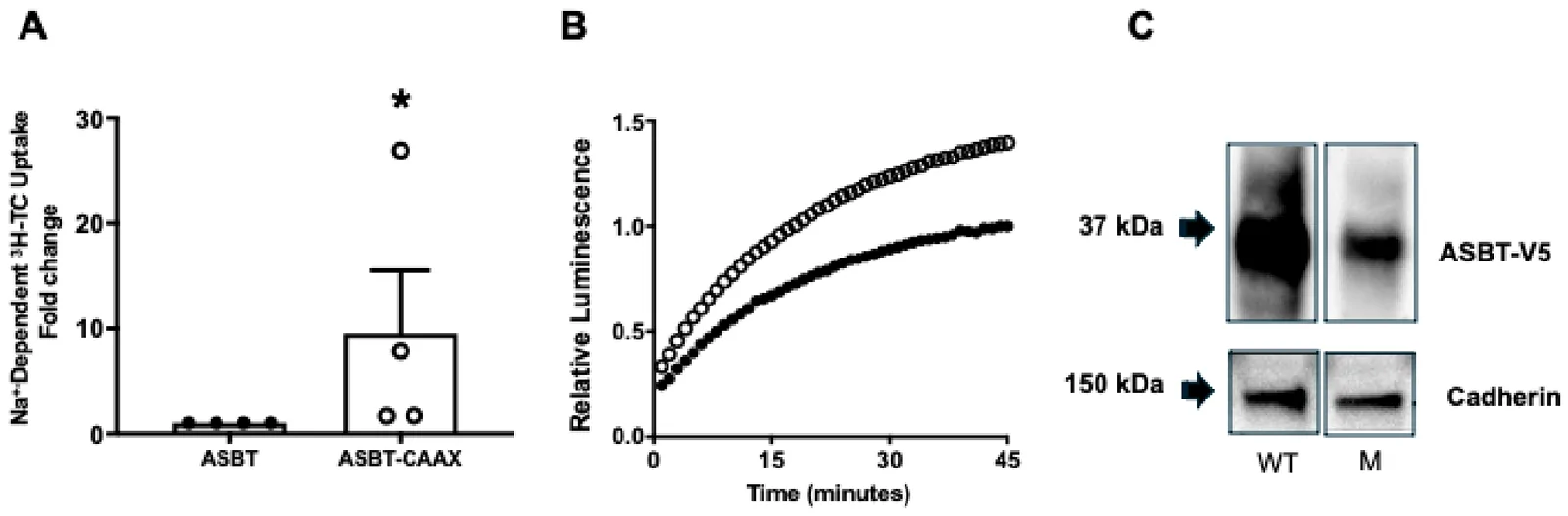
ASBT harboring a CAAX box at the C-terminus has increased transport activity. (**A**) HEK293 cells were transiently transfected with ASBT-V5 constructed with or without a CAAX box. ASBT function was assessed as Na^+^-dependent ^3^H-Taurocholic acid (TC) uptake normalized to ASBT-V5 protein expression. * *p* ≤ 0.05 as compared to ASBT without CAAX box. (**B**) A representative tracing of ASBT function with (open circles) or without CAAX box (closed circles) was evaluated by CA-SS-Luc bioluminescence in transiently transfected cells. (**C**) HEK293 cells were transiently transfected with ASBT-V5 (WT) or ASBT-V5-CAAX (M), and the levels of protein expression were assessed by Western blotting using anti-V5 antibodies and cadherin as an internal control.

We and other researchers previously showed that a pan S-acylation inhibitor reduced ASBT activity, suggesting that direct fatty acid binding to ASBT affects its function [14,15]. To further confirm that notion, we examined the impact of direct attachment of a lipid moiety to the ASBT protein on its function, mimicking the effects of lipidation via S-acylation. To that end, we designed an expression vector in which a CAAX box was added to the C-terminus of the ASBT-V5 fusion protein, downstream of the V5 tag (ASBT-V5-CAAX). The attached CAAX box was identical to that found in the human RAS gene (CCTGCAGGCTGCATGAGCTGCAAGTGTGTGCTCTCC) [17]. The CAAX box is a specific motif that undergoes prenylation, leading to the attachment of C-15 (farnesyl) or C-20 (geranylgeranyl) fatty acids to the cysteine residue and the generation of a hydrophobic C-terminus [17]. Indeed, this construct represents a CAAX-mediated prenylation of ASBT. Our findings showed that the basal activity of ASBT-V5-CAAX, as judged by the Na^+^-dependent ^3^H-TC uptake, is higher than ASBT-V5 (Figure 1A) in transiently transfected human kidney embryonic HEK-293 cells. Similarly, uptake of CA-SS-Luc by ASBT-V5-CAAX was higher than uptake by ASBT-V5 (Figure 1B). For both approaches, we normalized ASBT activity to total V5 expression to account for differences in transient transfection efficiency (Figure 1C). We further performed a cell-surface biotinylation to assess the abundance of ASBT-V5-CAAX on the plasma membrane. The results shown in Supplementary Figure S1 indicate that, similar to the total cellular protein expression, the abundance of the ASBT-VA-CAAX fusion protein on the plasma membrane, relative to membrane-associated cadherin, is markedly lower than that of the ASBT-V5 fusion protein. These data suggest that direct attachment of saturated fatty acid moieties to ASBT generally increases its basal activity.

### 2.2. ASBT Activity Is Decreased by EPA and DHA

**Figure 2:**
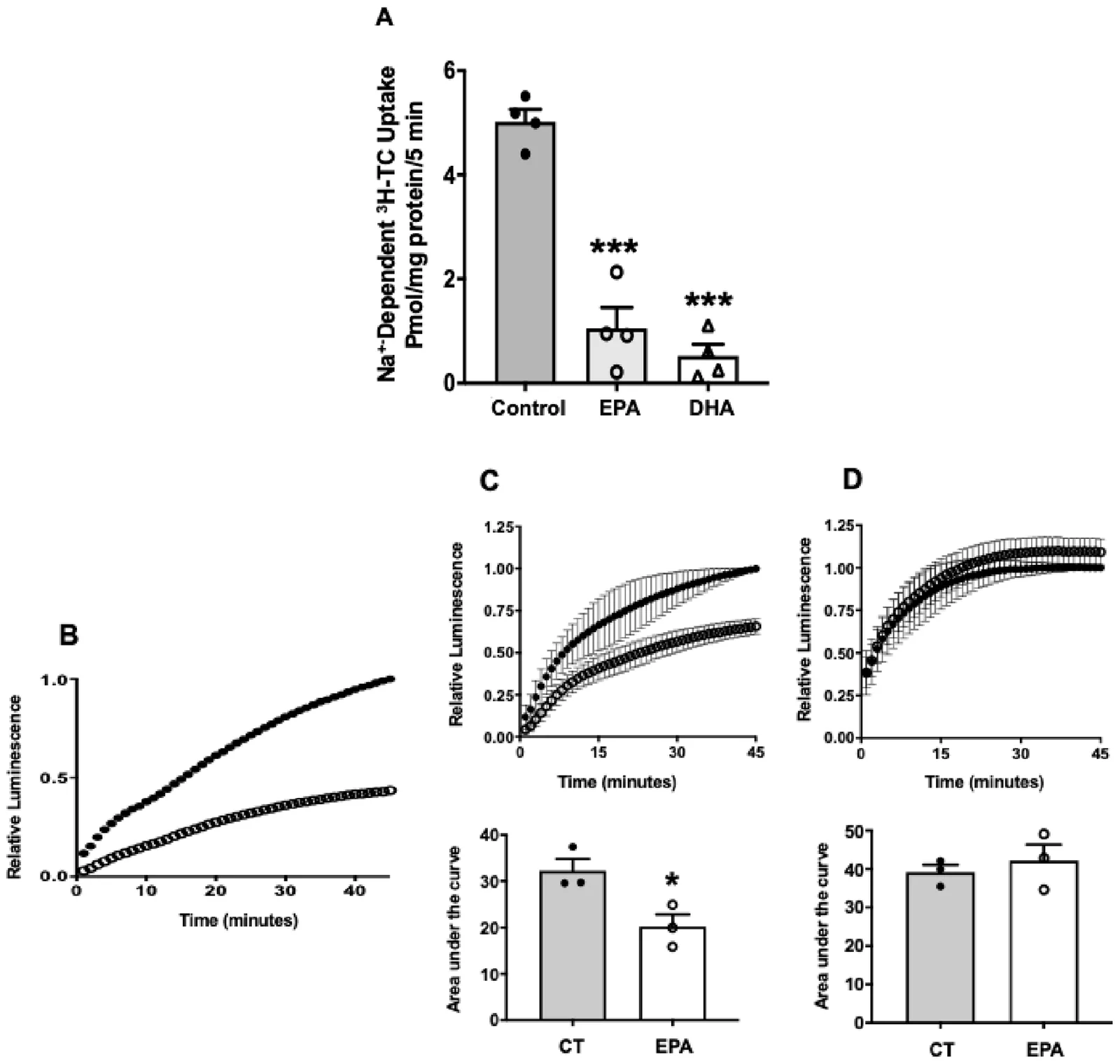

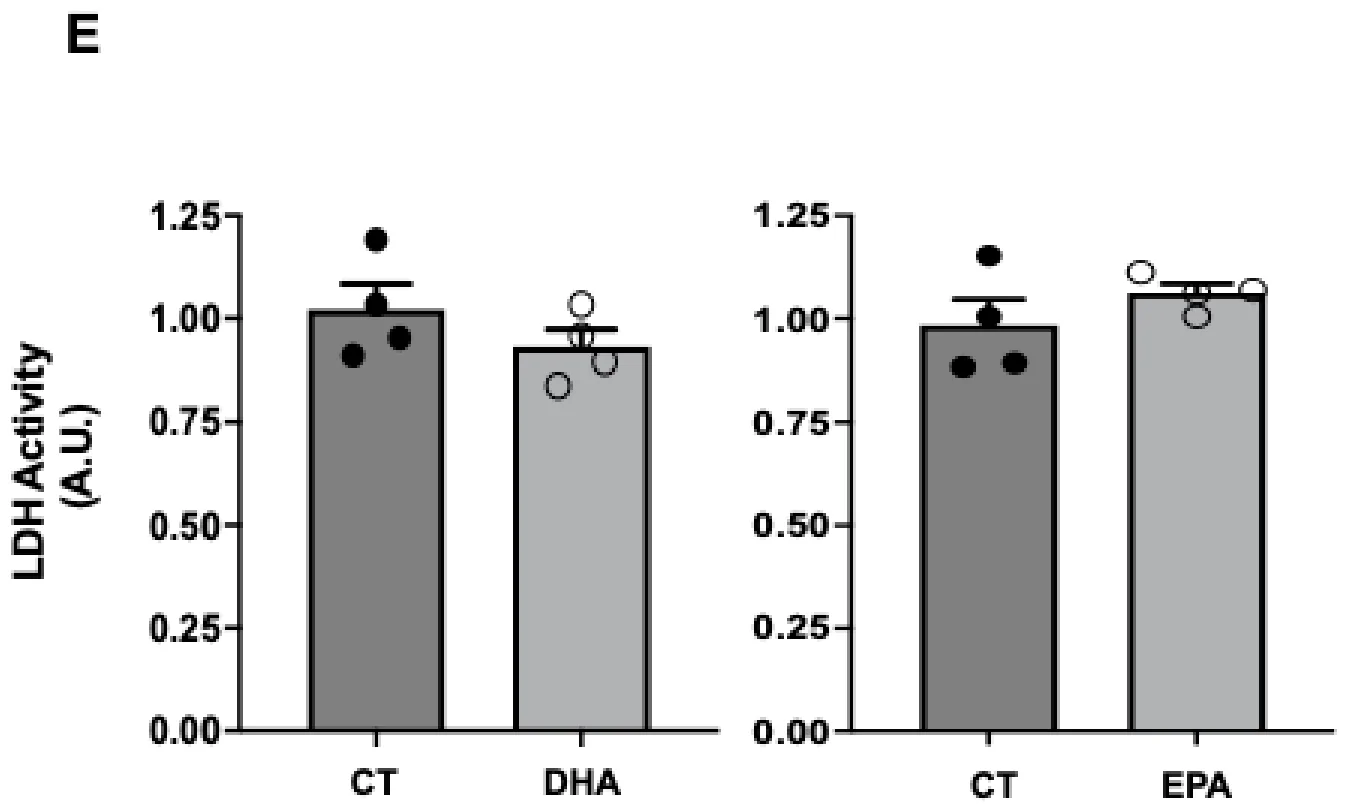
Effects of EPA and DHA on ASBT function in 2BT cells. (**A**) HEK293 cells stably transfected with ASBT-V5 fusion protein (2BT cells) were treated overnight with 100 mM of either EPA or DHA, and ASBT was then assessed as Na^+^-dependent ^3^H-Taurocholic acid (TC) uptake. *** *p* < 0.05 compared with untreated cells. (**B**) A representative tracing of ASBT function evaluated by the bioluminescence method as described in Methods in 2BT cells treated overnight with 100 mM DHA (open circles) or left untreated (closed circles). (**C**) ASBT function was assessed by the bioluminescence method in 2BT cells treated overnight with 100 mM of EPA (open circles) or left untreated (closed circles). Tracer signals for the bioluminescence are shown, and the bar graph shows the Area Under the Curve (AUC) values from three independent experiments. Data shown as Mean ± SE. * *p* < 0.05 as compared to untreated cells. (**D**) 2BT cells were treated with 100 mM EPA or left untreated overnight, and ASBT function was measured (1C). The media were then washed out, and the cells were left in control media with no EPA for an additional 24 h. At the end of the washout, ASBT function was reassessed using the bioluminescence method. Tracers of the bioluminescence signal are shown, and the bar graph shows the AUC values from three independent experiments. (**E**) Cells were treated for 24 h with 100 µM of DHA and EPA, and Lactate Dehydrogenase (LDH) activity was measured in the media as described in the Methods section. Data represent the Mean ± SE.

We further investigated the potential effects of omega-3 fatty acids, EPA and DHA, on ASBT function. Figure 2A shows that incubation of 2BT cells with 100 µM of DHA or EPA for 15 h resulted in a significant decrease in ASBT activity as assessed by measuring the Na^+^-dependent ^3^H-TC uptake. The effect was further confirmed via a bioluminescent assay, which demonstrated that DHA and EPA decreased ASBT activity (Figure 2B,C). Since the bioluminescent probe approach assesses ASBT function in living cells, we washed away the EPA-containing medium, allowed the cells to recover for 24 h, and then re-evaluated ASBT function. As shown in Figure 2D, ASBT activity was restored to control levels after washout, indicating that the initial decrease was unlikely to be due to cell death. This was further supported by measuring lactate dehydrogenase (LDH) activity as a surrogate for cell death, which showed no significant difference between the control and EPA- or DHA-treated cells (Figure 2E).

### 2.3. Supplementation of Fish Oil as a Source for n-3 PUFAs Alters Intestinal Handling of Bile Acids

**Figure 3:**
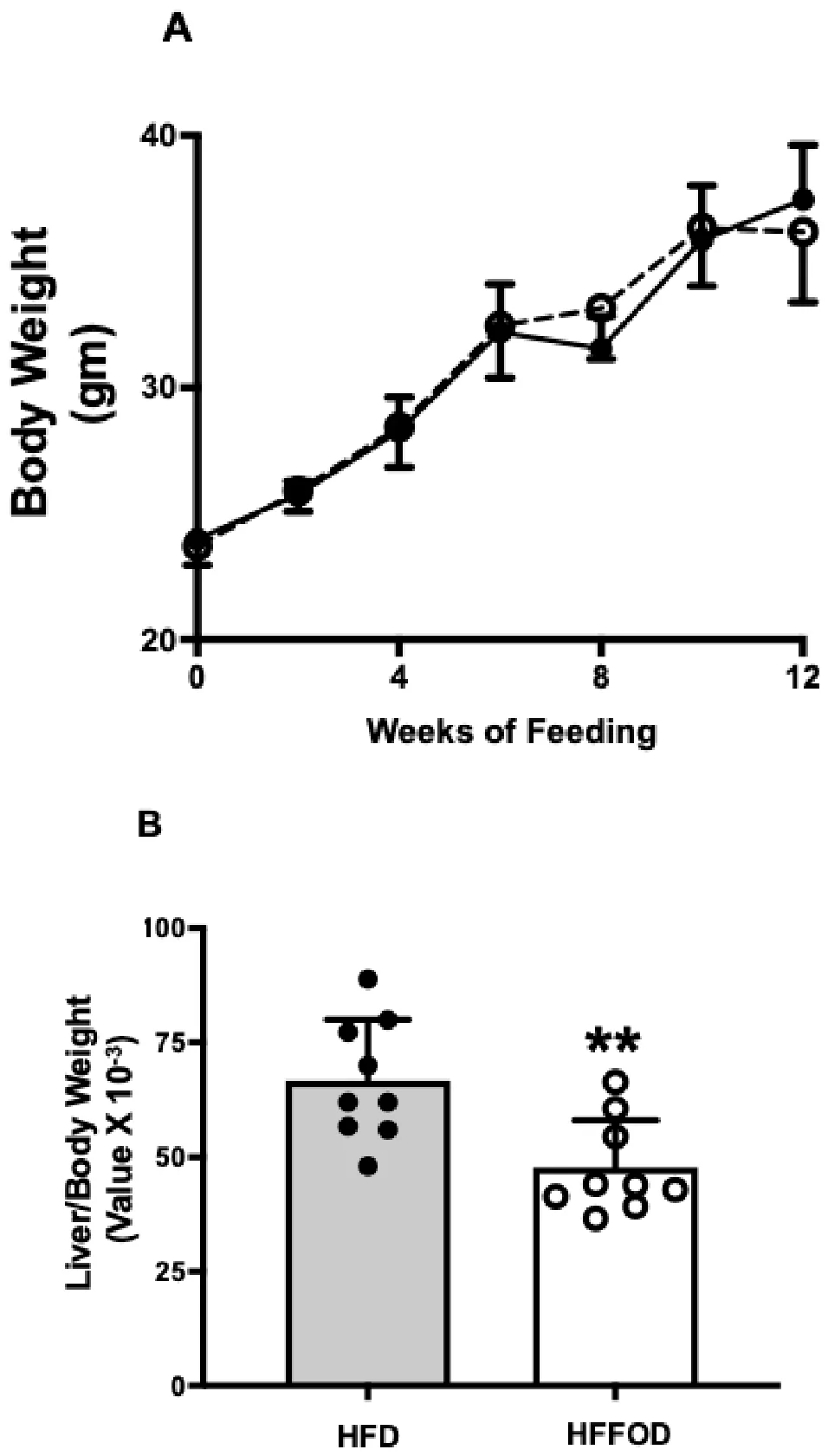
Effects of fish oil supplementation to a high-fat diet (HFD) on liver and body weight in mice. Mice were fed a high-fat diet as described in Methods (HFD) or a high-fat diet supplemented with fish oil (HFFOD). (**A**) body weight over the course of feeding, results presented as mean ± SD. (**B**) Liver-to-body weight ratios. ** *p* < 0.05 as compared to HFD.

**Figure 4:**
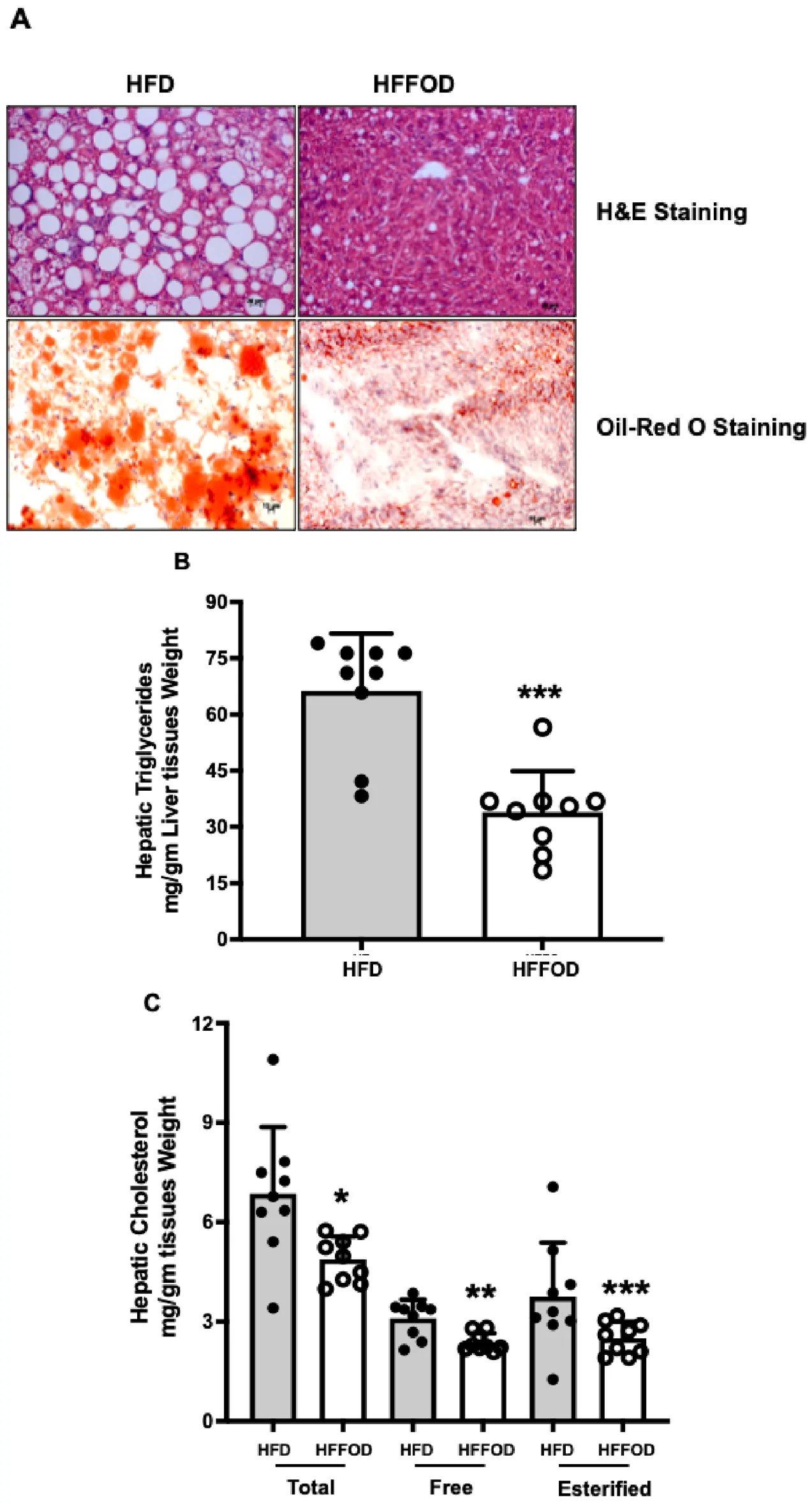

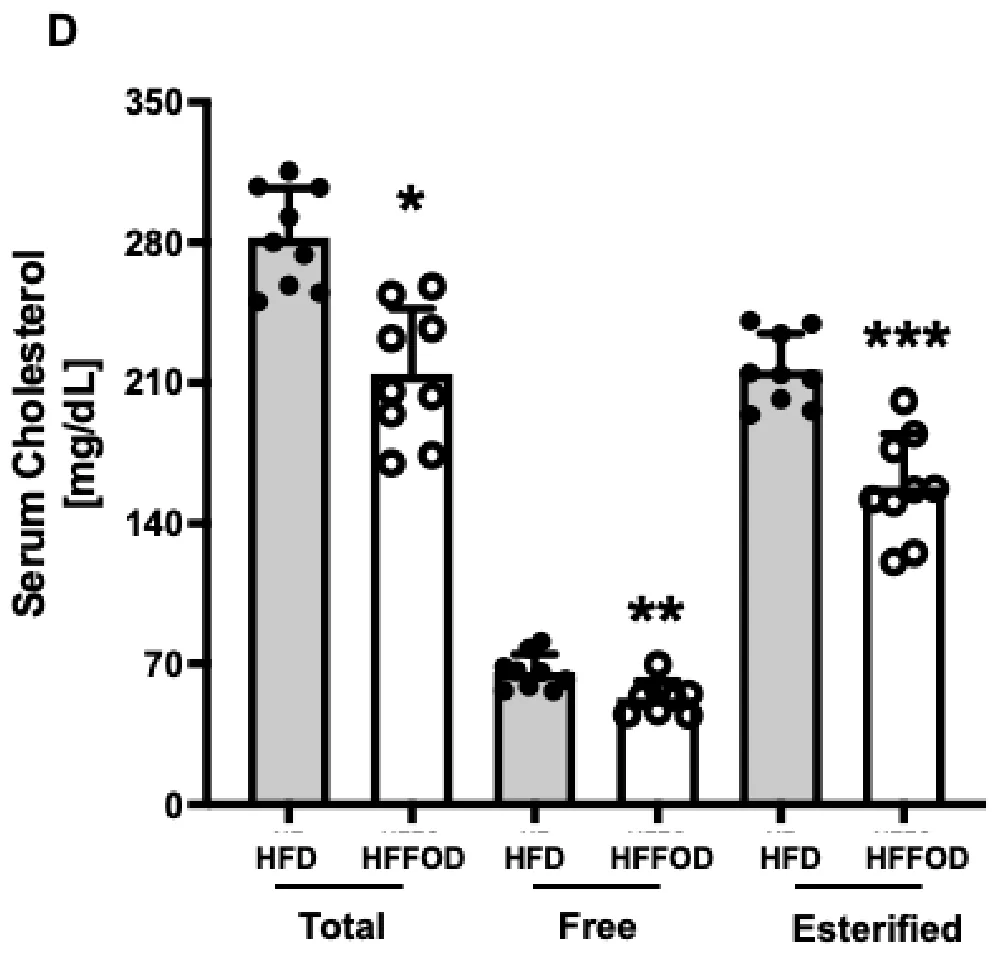
Effects of fish oil supplementation on diet-induced hepatic steatosis. (**A**) Liver sections from mice fed a high-fat diet (HFD) or a high-fat diet supplemented with fish oil (HFFOD) stained with hematoxylin–eosin (H&E) or oil red O to visualize hepatic lipid droplets. (**B**) Hepatic levels of triglycerides in mice fed an HFD or HFFOD. *** *p* < 0.05 as compared to HFD. (**C**) The levels of total, free, and esterified cholesterol in mice fed with either HFD or HFFOD. (**D**) Serum levels of cholesterol in mice fed HFD or HFFOD. *, **, *** *p* < 0.05 for total, free, and esterified cholesterol, respectively, as compared to HFD.

**Figure 5:**
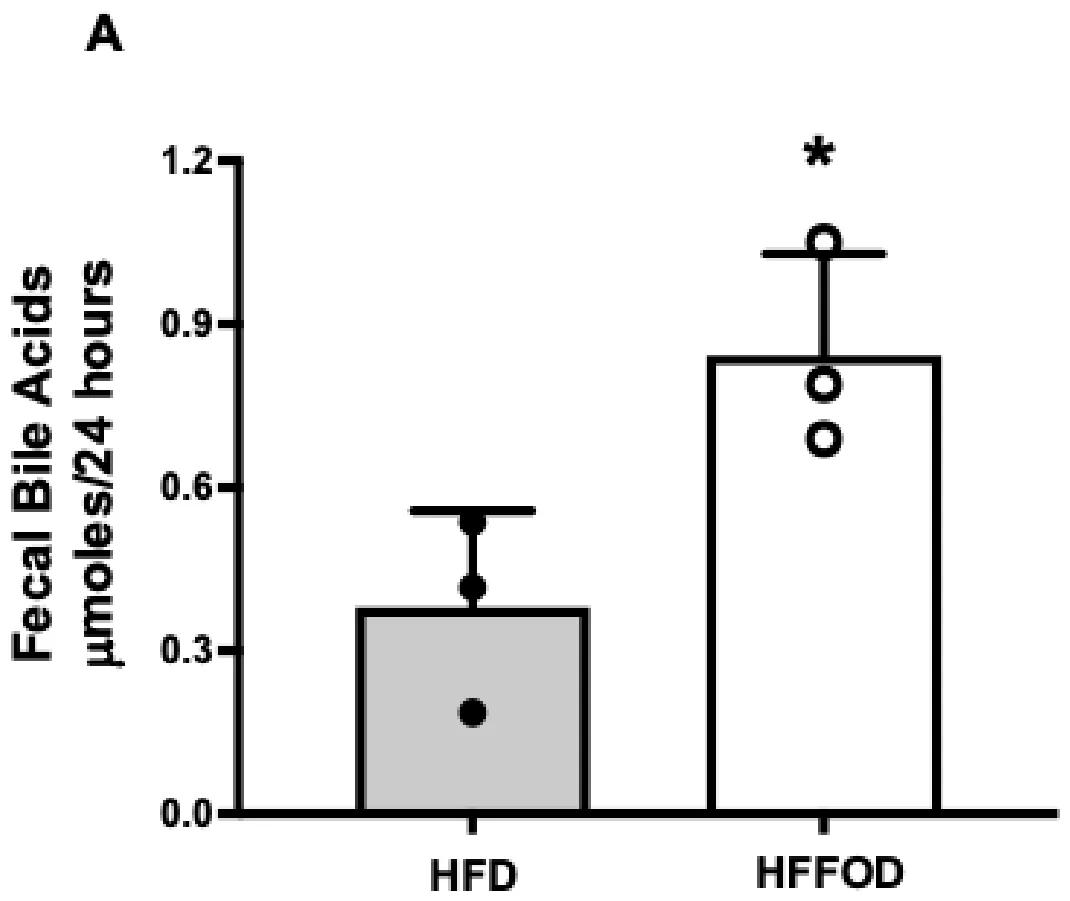

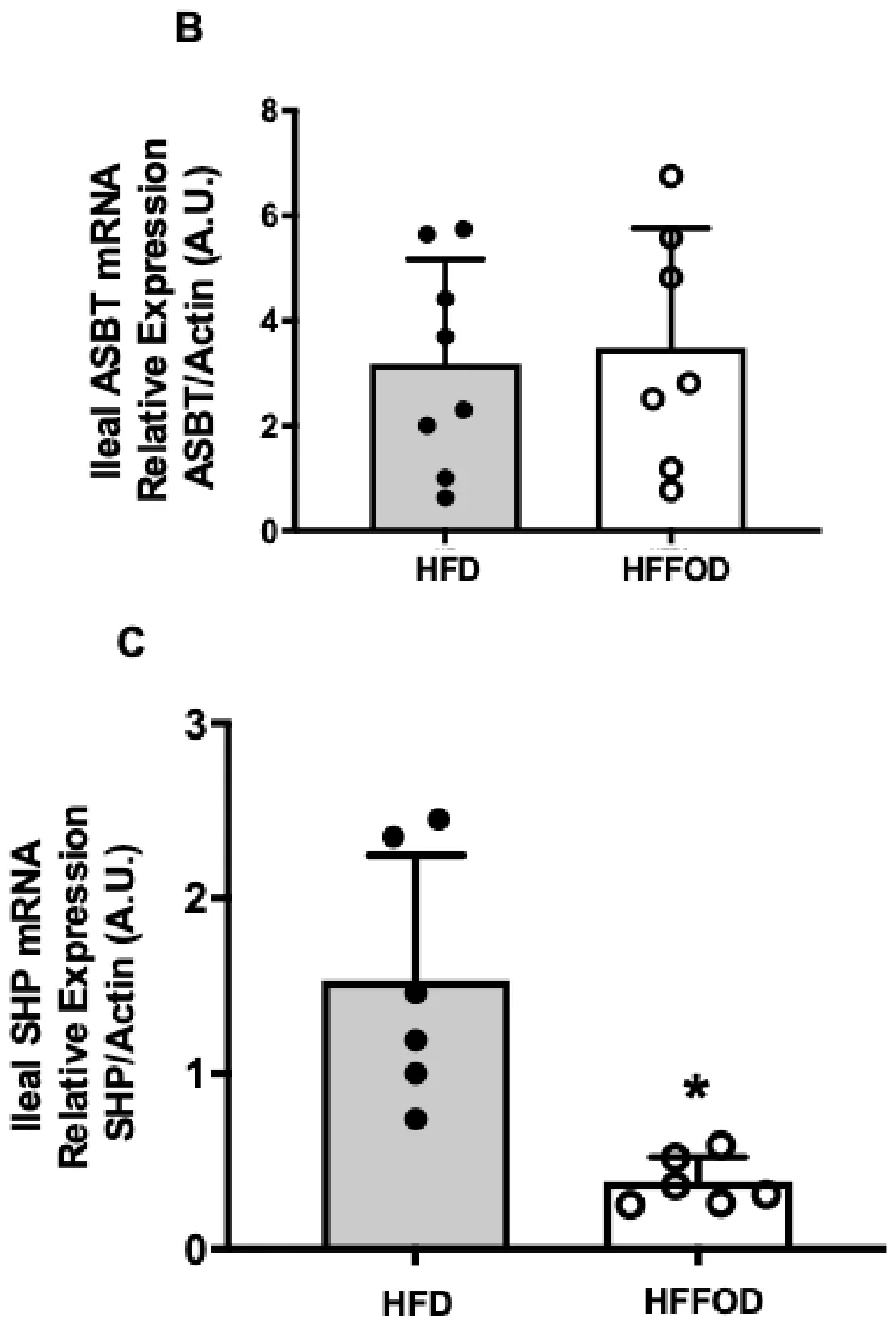
Effects of fish oil supplementation on Ileal bile acid absorption. (**A**) Fecal levels of bile acids extracted from mice fed a high-fat diet or a high-fat diet supplemented with fish oil as described in Methods. * *p* < 0.05 as compared to HFD. (**B**) The relative expression of the ileal ASBT was assessed by real-time PCR in mice fed either HFD or HFFOD (A.U.: Arbitrary Unit). (**C**) The relative expression of the ileal short heterodimer (SHP) was assessed by real-time PCR in mice fed either HFD or HFFOD (A.U.: Arbitrary Unit). * *p* < 0.05 as compared to HFD.

**Figure 6:**
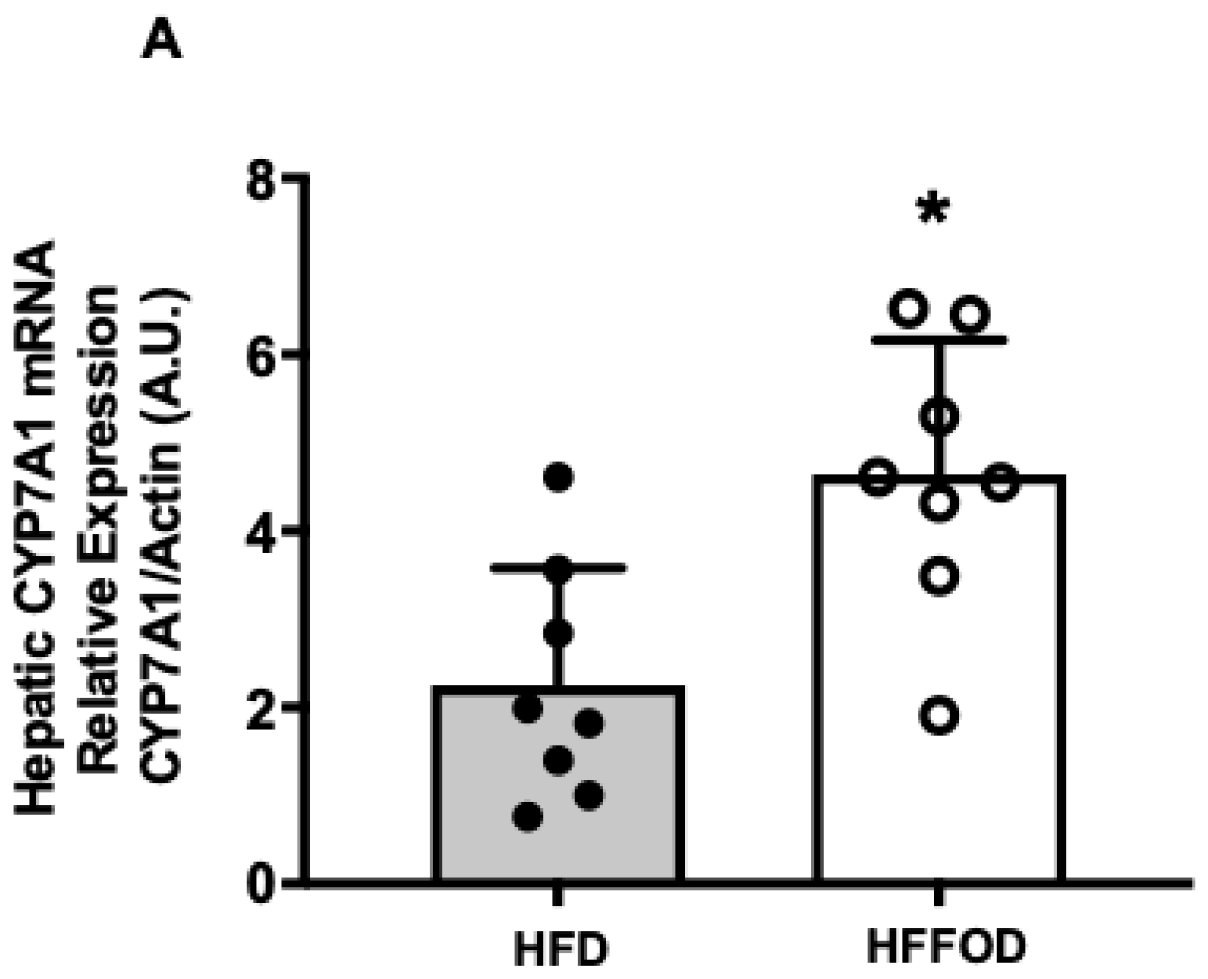

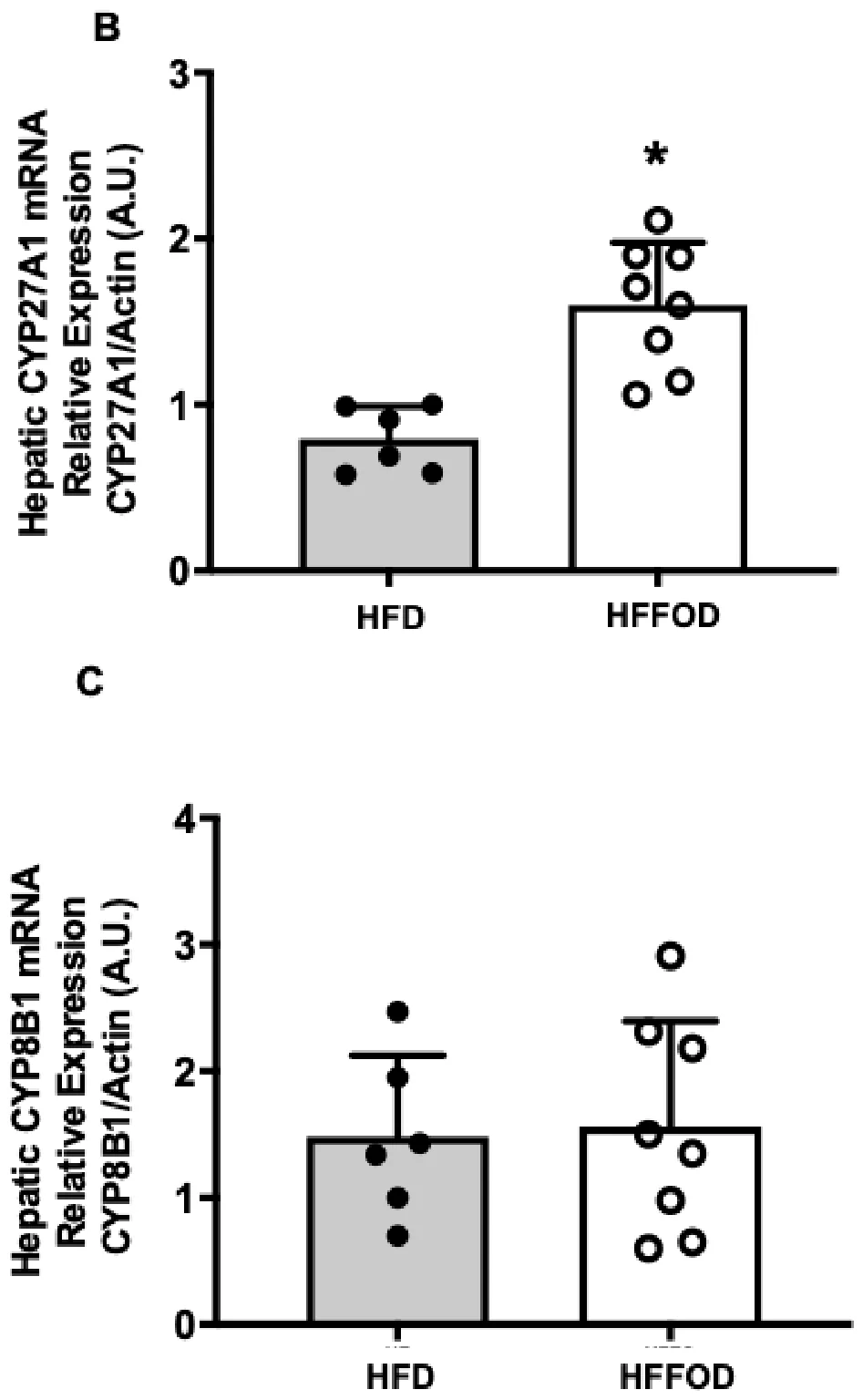
Effects of fish oil supplementation on gene expression of hepatic bile acid synthesis. The relative expression of hepatic genes involved in bile acid synthesis in mice fed either a high-fat diet (HFD) or a high-fat diet supplemented with fish oil (HFFOD). (**A**) The relative expression of hepatic CYP7A1. (**B**) The relative expression of hepatic CYP27A1. (**C**) The relative expression of hepatic CYP8B1. A.U.: Arbitrary Unit; * *p* < 0.05 as compared to HFD.

We next aimed to examine the effects of feeding n-3 PUFAs supplemented to mice as fish oil, along with a high-fat and high-cholesterol diet (HFD), on intestinal bile acid absorption and hepatic steatosis. First, we investigated changes in body and liver weights during the feeding. As shown in Figure 3A, fish oil supplementation did not alter the changes in body weight as compared to the HFD diet alone. However, fish oil supplementation significantly reduced the liver-to-body weight ratio (Figure 3B). Supplementation of fish oil to the HFD diet decreased hepatic steatosis, as assessed by H&E and red O-oil staining (Figure 4A). Fish oil supplementation also decreased hepatic triglyceride levels (Figure 4B), hepatic cholesterol levels (Figure 4C), and serum cholesterol (Figure 4D). Regarding bile acids, our findings showed that fish oil supplementation increased fecal bile acids, suggesting decreased bile acid absorption (Figure 5A), without altering ileal ASBT mRNA (Figure 5B). The expression of ileal small heterodimer partner (SHP) mRNA, which is a target of the bile acid receptor FXR, was decreased, further supporting the notion of a decrease in bile acid absorption (Figure 5C). Concomitant with this observation, hepatic CYP7A1 mRNA expression, the key enzyme in bile acid synthesis, was increased in response to fish oil, indicating that the observed reduction in bile acid absorption attenuated the negative feedback effect on bile acid synthesis (Figure 6A). Also, the expression of hepatic CYP27A1, which is critical for bile acid synthesis, was increased with the supplementation of fish oil (Figure 6B). However, CYP8B1 expression, the enzyme responsible for cholic acid synthesis, remained unaltered (Figure 6C), suggesting specific effects on bile acid synthesis enzymes. Collectively, our data indicate that supplementing a high-fat diet with omega-3 fatty acids decreased ASBT activity, unlikely due to altering its ileal protein levels. Such a decrease in ASBT function triggers a compensatory increase in hepatic bile acid synthesis, which could explain the observed reductions in hepatic and serum cholesterol levels.

### 2.4. Direct Binding of EPA to ASBT

**Figure 7:**
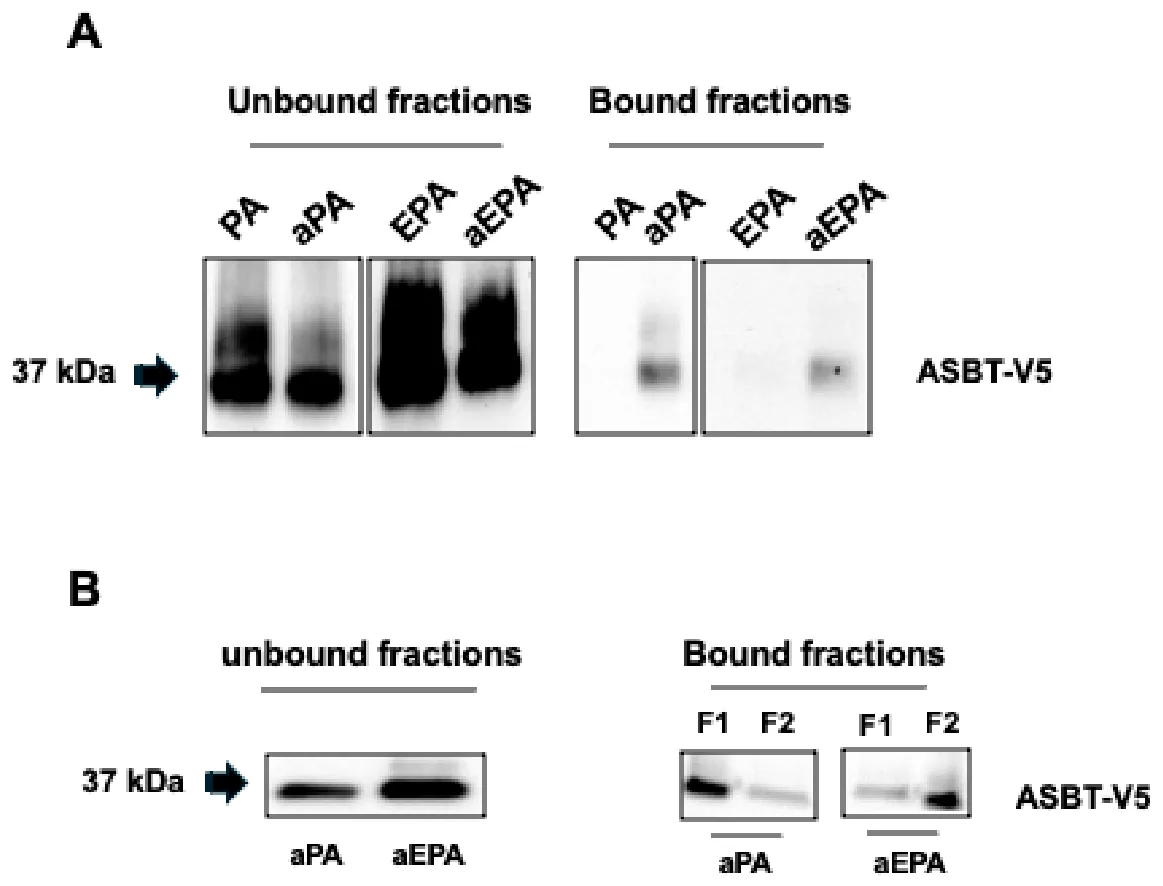
Metabolic labeling with alkyne-palmitate and alkyne EPA. (**A**) 2BT cells were incubated with alkyne palmitate (aPA) or alkyne-EPA (aEPA) for 12 h and cells were then harvested. Alkyne fatty acids incorporated into proteins are then conjugated to biotin via click chemistry. Biotinylated proteins are precipitated with NeutrAvidin agarose resin. The blots show the incorporation of aPA and aEPA in ASBT. (**B**) The cells were labeled with alkyne fatty acids as indicated, and then the agarose resin was first treated with hydroxylamine, and the eluted proteins were designated as fraction 1 (F1). The remaining proteins on the agarose resin were then eluted into Laemmli buffer and designated as fraction 2 (F2). In the case of aPA, the majority of ASBT bound to aPA was eluted in F1, indicating a binding via S-acylation. In the case of aEPA, ASBT was mainly eluted in F2, indicating a binding that is largely independent of S-acylation.

To gain mechanistic insights into EPA’s effects on bile acid absorption, we examined whether EPA directly binds ASBT. Figure 7A shows direct binding of alkyne EPA to ASBT, as assessed by metabolic labeling of HEK293 cells followed by a click reaction with azide-biotin and pull-down with avidin beads (alkyne palmitate used as a positive control). These findings suggest that the effect of EPA-mediated decrease in bile acid uptake could result from direct binding to ASBT. Furthermore, Figure 7B shows that retrieval of ASBT bound to alkyne-EPA was largely resistant to hydroxylamine treatment, indicating that the binding occurs via a mechanism distinct from S-acylation. Yet, S-acylation appears to mediate the direct attachment of alkyne palmitate (Figure 7B), as binding was highly sensitive to hydroxylamine-mediated retrieval. However, the responsible DHHC acyltransferase remains elusive.

### 2.5. The Expression of Intestinal DHHC Acyltransferases

**Figure 8:**
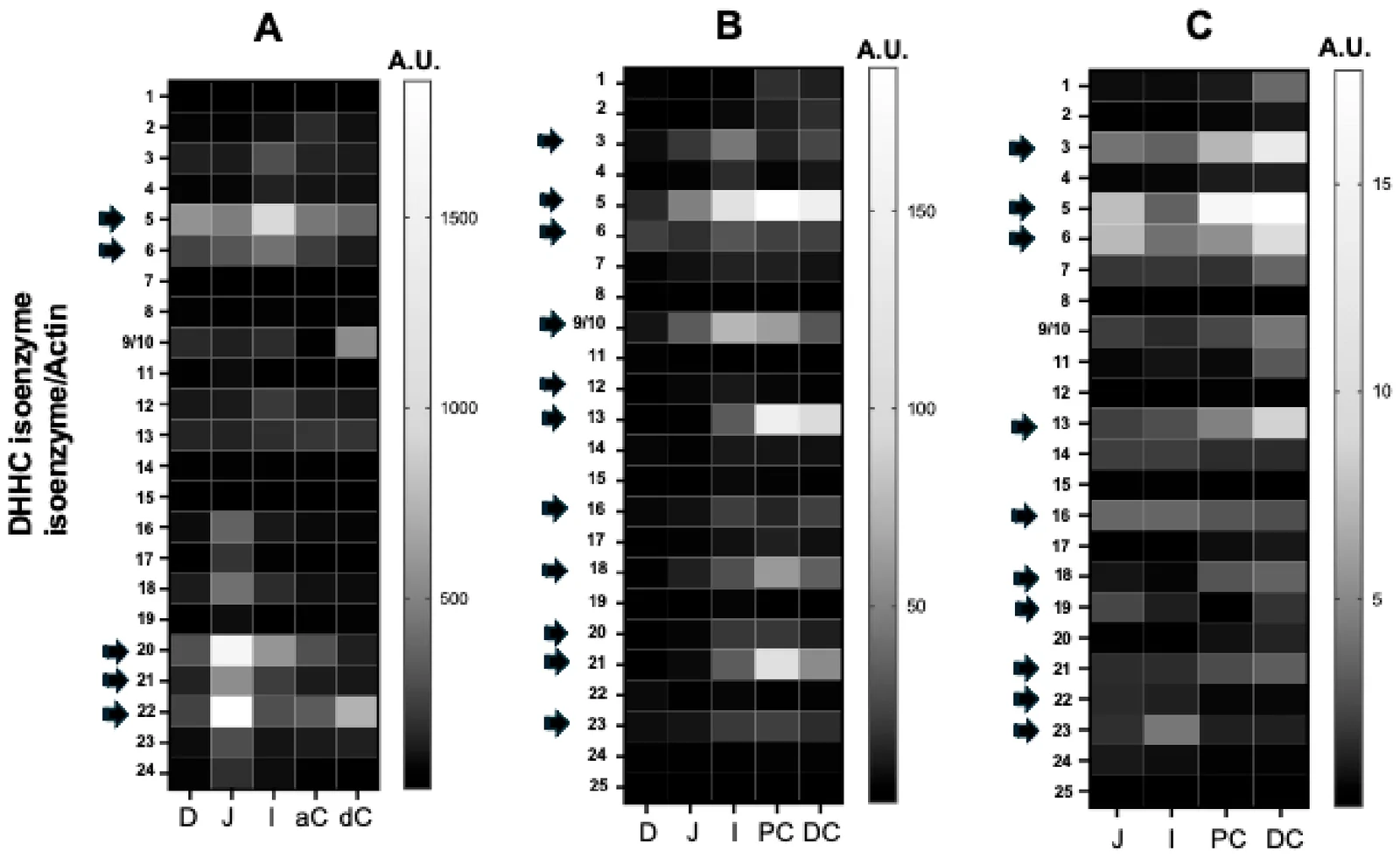
The relative expression of DHHC isoenzymes in different segments of the intestine and colon. Total RNA was extracted from different segments of the small intestine and colon, and gene expression was assessed by real-time PCR using gene-specific primers. (**A**) Expression of DHHCs in the mucosa of different segments of the human (**A**) and mouse (**B**) intestine and colon. (**C**) DHHCs’ relative expression from RNA extracted from isolated epithelial cells of the mouse small intestine and colon. The values are expressed relative to an arbitrary unit (A.U.) set to 1. D: Duodenum, J: Jejunum; I: Ileum; aC: ascending colon; dC: descending colon; PC: Proximal colon; DC: Distal colon.

S-acylation is mediated by a large family of acyltransferases containing the DHHC domain (Asp-His-His-Cys), namely DHHC1-24, including DHHC25 in mice [16]. First, we examined the relative mRNA expression of DHHCs along the GI tract. Figure 8 depicts the relative expression of DHHCs in the human GI tract (Figure 8A) and mouse GI tract (Figure 8B). We also investigated the relative expression of DHHCs in intestinal epithelial cells isolated from different segments of the mouse GI tract, and the data are shown in Figure 8C. The values shown in the figures are expressed relative to an arbitrary unit (A.U.) set to 1. In the human and mouse intestine, DHHC3, C5, C6, C12, C15, C17, C18, C20, and C21 appear to be relatively abundant in various regions of the GI tract. Overall, the data demonstrate that each DHHC enzyme exhibits differential expression patterns across various intestinal and colonic segments. Furthermore, the expression of most DHHCs in isolated intestinal epithelial cells follows a pattern similar to that observed in the mucosa.

### 2.6. DHHC21 Is Involved in ASBT S-Acylation

**Figure 9:**
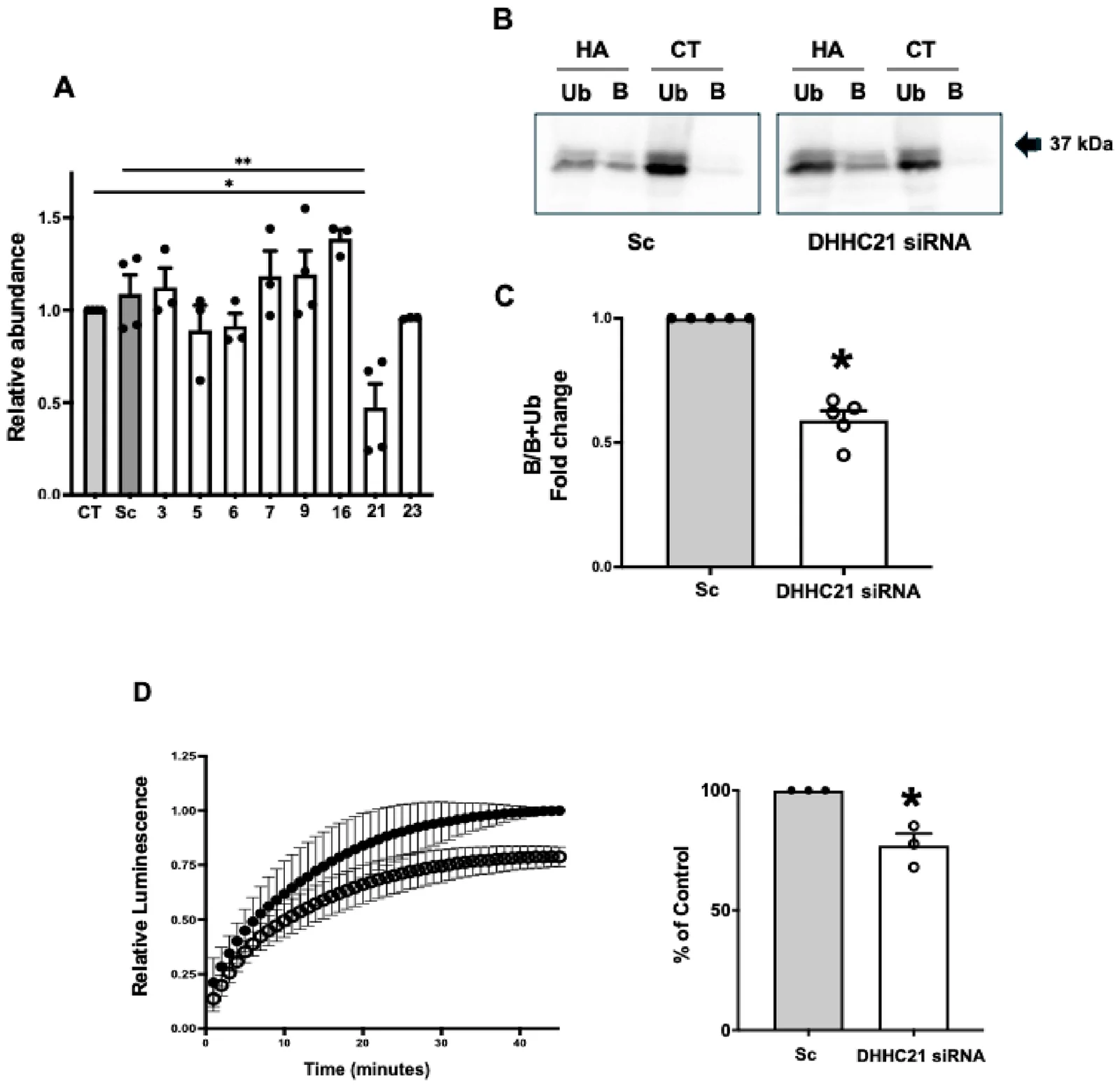
The attenuation of DHHC21 decreases ASBT S-acylation and transport activity. (**A**) Transfection of 2BT cells (239 cells stably transfected with ASBT-V5 fusion protein) with siRNA specific to DHHC21 decreased the expression of DHHC21 but not the expression of other DHHC isoenzymes. * *p* < 0.05 from control (not transfected) cells, ** *p* < 0.05 from cells transfected with scramble siRNA (Sc). (**B**) A representative Western blot from an acyl-RAC experiment demonstrating a decrease in the bound fraction (**B**) of ASBT-V5 relative to the unbound fraction (Ub) in cells transfected with siRNA specific for DHHC21 after blocking free thiol sites and treatment with protein lysates with hydroxylamine (HA). CT represents samples that were not treated with HA before pulling down the S-acylated proteins with the beads. (**C**) Densitometric analysis demonstrating the decrease in the ratio of bound to unbound ASBT-V5 from several acyl-RAC experiments. * *p*< 0.05 compared to cells transfected with scramble siRNA (Sc). (**D**) ASBT function is decreased by the reduction in DHHC21 expression. Cells were transfected with DHHC21-specific siRNA or scramble siRNA, and ASBT activity was assessed using the bioluminescent bile acid probe and IVIS system as described in Methods. The graphs represent the bioluminescent signal from cells transfected with DHHC21-specific siRNA (open circles) or scramble siRNA (closed circles), and the area under the curve from signals obtained from DHHC21-attenuated cells was significantly less than that from cells transfected with scramble siRNA (Sc). * *p* < 0.05 as compared to Sc.

Next, we investigated the DHHC isoenzyme involved in ASBT S-acylation and initially focused on DHHC21, as it has previously been shown to regulate intestinal barrier function [18]. As shown in Figure 9A, siRNA targeting DHHC21 specifically reduced DHHC21 mRNA expression and did not affect the expression of other isoenzymes. Figure 9B,C indicate that attenuation of the DHHC21 isoenzyme significantly decreased ASBT S-acylation, as assessed by the Acyl-RAC approach. Concomitant with the decline in ASBT S-acylation, lowering DHHC21 expression also reduced ASBT-V5 function as assessed by the bioluminescent approach (Figure 9D). These observations strongly suggest that DHHC21 is involved in the S-acylation of ASBT and in maintaining its transport activity.

## 3. Discussion

We and other researchers have shown that the ileal bile acid transporter ASBT is S-acylated at the basal level and that its transport activity is decreased by an S-acylation inhibitor [14,15]. Recent studies have shown that another bile acid transporter expressed on the basolateral membrane of intestinal epithelial cells, the Organic Solute Transporter alpha (Osta) is also S-acylated with fatty acids and that such acylation is critical for its transport function [19]. These collective findings suggest that modulation by fatty acids and S-acylation are regulatory processes critical for intestinal bile acid absorption.

S-acylation is catalyzed by acyltransferase enzymes that harbor a conserved aspartate-histidine-histidine-cysteine-rich domain (DHHCs) and belong to a large family of isoenzymes, including 23 members in humans and 24 in mice [16,20]. DHHC isoenzymes have been shown to exhibit differential cellular localization and substrate specificity for lipids and target proteins [20], but their distribution along the length of the intestine and colon remains unknown. Our data showed that the DHHC5 isoenzyme is the most abundantly expressed DHHC in human and mouse intestinal mucosa and in isolated mouse intestinal epithelial cells. DHHC5 has been shown to be associated with the plasma membrane and endocytic vesicles and to be a crucial regulator of the CD36 fatty acid transporter [21]. Yet DHHC5 likely plays other functional roles in the gut that remain to be investigated. Other DHHCs that appear to be abundant in human and mouse intestinal mucosa and in isolated intestinal epithelial cells include DHHC3, 6, 9, 11, 15, 16, 17, 19, 20, 21, and 22. Mutations in humans and deletions in mice of several of these DHHCs have been associated with liver disorders and pancreatic cancer [20], suggesting their crucial roles in maintaining the function of organs involved in the GI system.

To identify the DHHC enzyme involved in ASBT S-acylation, we focused on DHHC21 in the current study, as it has previously been shown to modulate intestinal barrier function [18]. Using siRNA and Acyl-RAC approaches, our results strongly suggest that DHHC21 is involved in ASBT S-acylation. This was further supported by the decrease in ASBT function observed upon siRNA-mediated attenuation of DHHC21 expression. DHHC21 was previously shown to function as a Ca^2+^-calmodulin-dependent acyltransferase critical for CD4+ T cell activation [22]. Mice expressing functionally deficient DHHC21 were also shown to have attenuated renal dysfunction in response to sepsis [23] and, in other reports, to protect endothelial integrity in a model of systemic inflammation [24]. Prior studies showed that proinflammatory cytokines stimulated DHHC21 expression in intestinal epithelial cells and that mice expressing a dysfunctional mutant of DHHC21 were protected against burn injury-induced gut barrier dysfunction [18]. Our findings add novel insights into the functional roles of DHHC21 in the intestine, showing its involvement in ASBT S-acylation and bile acid absorption.

Our current findings with the ASBT prenylation mimetic strongly suggest that direct attachment of a saturated C15 or C20 fatty acid to ASBT markedly increases its activity. Prenylation is a process that is initiated by the attachment of geranylgeranyl (C20) or farnesyl (C15) to the cysteine residue C in the CAAX motif, followed by cleavage of the AAX sequence and the formation of a hydrophobic C-terminus [17]. It was noted that the total levels of ASBT-V5 protein tagged to CAAX were less than those of the untagged protein. This could reflect decreased transfection efficiency or changes in protein stability. Previous studies have shown that prenylation alters protein stability [25], and tagging the CAAX box may have decreased ASBT protein stability.

We have previously shown that ASBT function is increased by the saturated fatty acid palmitate and decreased by the monounsaturated fatty acid oleate [14]. Our novel data from the current studies show that the omega-3 polyunsaturated fatty acids DHA and EPA directly affect ASBT function, reducing its transport activity. We also provided novel evidence that EPA directly binds ASBT. Notably, our findings showed that EPA binding to ASBT was resistant to cleavage by hydroxylamine, suggesting a binding mechanism different from S-acylation. Indeed, recent studies have shown that EPA forms methylene bridges that acylate the delta1 carbon of tryptophan residues on PDK1, promoting its recruitment to the membrane and activation of PI3K activity and glucose uptake [26]. Future studies should explore the potential roles of tryptophan residues in ASBT in binding EPA. Collectively, it appears that different types of fatty acids can bind ASBT, influencing its transport activity and affecting bile acid absorption in a way dependent on their degree of saturation. These observations have significant implications. It is plausible that the increase in ASBT function in response to dietary overload of saturated fatty acids, such as palmitate, is an adaptive response whereby S-acylation to palmitate increases ASBT activity and bile acid absorption, leading to an expanded bile acid pool and enhanced emulsification capacity to promote palmitate absorption. In contrast, ASBT binding to n-3 PUFAs reduces bile acid absorption and likely decreases the bile acid pool, which may, at least in part, mediate their beneficial effects on plasma cholesterol levels and hepatic steatosis.

We investigated the impact of dietary supplementation with fish oil, a source of EPA and DHA, in a model of diet-induced hepatic steatosis to directly examine bile acid modulations in a disease context rather than in response to a control diet. Our findings clearly showed that fish oil supplementation decreased hepatic lipid accumulation independently of weight loss, indicating the specificity of its effects on steatosis. Notably, fish oil supplementation increased the expression of hepatic enzymes responsible for bile acid synthesis. This is consistent with previous studies showing that dietary fish oil induces bile acid synthesis in humans [27] and mice [28]. We should note that improvements observed in animals fed n-3 PUFAs could arise from reduced exposure to the saturated fats, a direct action of n-3 PUFAs, or both, as indicated in prior studies [29]. It is also important to mention that n-3-PUFAs have been shown to modulate various aspects of lipid metabolism, including suppressing fatty acid synthesis [30], increasing β-oxidation [31], and reducing VLDL secretion and blood TG levels, as well as their accumulation in the liver [32]. Also, recent evidence suggests that n-3-PUFAs may reduce intestinal fat absorption by modulating lacteal permeability and chylomicron transport [33]. Additionally, n-3-PUFAs may affect several aspects of cholesterol homeostasis, including a decrease in cholesterol absorption [34] and a reduction in cholesterol synthesis. It is clear, therefore, that the beneficial effects of dietary fish oil supplementation on cholesterol and lipid metabolism are multifactorial. Our current findings support a decreased FXR target gene expression in the ileum, which indicates lower intracellular bile acid levels in ileal enterocytes, along with elevated fecal bile acid levels, together suggesting decreased bile acid absorption, followed by increased hepatic bile acid synthesis due to a reduced ileal FXR-mediated negative feedback inhibition. One limitation of our study is that the rates of bile acid synthesis and absorption, as well as the size of the circulating pool of bile acids, were not directly measured. Importantly, the increase in CYP7A1 is a compensatory response to the decrease in absorption. Our data do not allow us to determine whether this elevation in CYP7A1 expression increased bile acid synthesis to a level sufficient to expand the bile acid pool, as the size of the circulating bile acid pool was not measured in this study. The conclusion remains rather speculative, and future study will examine the extent to which n-3 PUFA-mediated reduction in bile acid absorption contributes to fat absorption and the protection against hepatic steatosis.

ASBT expression in response to dietary fish oil was unlikely to change, consistent with a post-translational modulation. The collective evidence from the current in vitro and in vivo experiments strongly suggests that EPA and DHA decrease ASBT activity by post-translational mechanisms. Our data suggest that direct binding of EPA and DHA to ASBT may be responsible for the decrease in its transport activity. Other potential mechanisms include altering the composition of plasma membrane lipid rafts [35]. Indeed, prior studies showed that ASBT activity is sensitive to the protein’s localization within lipid-raft microdomains of the plasma membrane [36]. EPA incorporation into the plasma membrane is likely to remain stable for 24 h after washout [37]. Since our data show that EPA effects on ASBT function are reversed by an overnight washout, and since ASBT has a short half-life [38], it is very likely that the observed reduction in function is due to direct binding to ASBT rather than to alterations in the composition of the membrane lipid raft.

## 4. Materials and Methods

### 4.1. Cell Culture and Materials

Human embryonic kidney 293 cells (HEK 293) were obtained from ATCC and maintained at 37 °C in a 5% CO_2_ atmosphere. The cells were grown in Dulbecco’s Modified Eagle Medium containing 4 mM L-glutaminutese, 4.5 g/L glucose, 1 mM sodium pyruvate, and 1.5 g/L sodium bicarbonate, supplemented with 10% fetal bovine serum, 100 units/mL penicillin, and 100 mg/mL gentamicin (Invitrogen, Carlsbad, CA, USA). HEK 293 cells stably transfected with human ASBT-V5 (2BT) were cultured in the same medium containing 7 μg/mL puromycin, as previously described [14]. All chemicals and reagents were purchased from Sigma-Aldrich (St. Louis, MO, USA) unless otherwise specified.

### 4.2. RNA Extraction and Quantitative Reverse Transcription PCR Analysis

For human tissues, total RNA was extracted from mucosal scrapings of the entire small intestine and colon of healthy adult organ donors, obtained from Gift of Hope (IL) immediately after organ harvest for transplantation (the samples were a generous gift from Dr. Pradeep Dudeja’s laboratory at the University of Illinois Chicago). For mouse tissues, RNA was extracted from liver, small intestinal, and colonic scrapings, and from isolated intestinal epithelial cells prepared as previously described [2]. Total RNA was extracted from liver tissues utilizing the RNeasy Mini kit (Qiagen, Valencia, CA, USA) according to the manufacturer’s instructions. Equal amounts of RNA from different samples were reverse-transcribed and amplified in a one-step reaction utilizing Brilliant SYBR Green QRT-PCR Master Mix Kit using gene-specific primers (Agilent Tech., Santa Clara, CA, USA). Results are expressed as relative gene expression, normalized to internal control genes (GAPDH). All primer sequences are listed in Table 1.

### 4.3. Western Blot Analysis

Cell surface biotinylation was performed as previously described [36]. For cellular protein extraction in other experiments, cells were scraped from the culture flasks into 1X cell lysis buffer (Cell Signaling Technologies, Danvers, MA, USA) with 0.1% SDS. Samples were then boiled for 5 min in Laemmli buffer (BioRad, Hercules, CA, USA) with 2.5% β-mercaptoethanol. The protein samples were resolved by SDS-PAGE and then transferred to nitrocellulose membranes. Specific proteins were probed utilizing antigen-specific antibodies. Anti-V5 tag antibodies were obtained from Invitrogen (catalog # 46-0705), anti-GAPDH antibodies were obtained from Sigma (catalog # G9545), and anti-ASBT antibodies were obtained from Santa Cruz (catalog # C1821).

### 4.4. Acyl Resin-Assisted Capture (Acyl-RAC)

Acyl-RAC was performed as previously described by us [14]. Free thiols in the samples were blocked by 1.5% S-methyl methanethiosulfonate (MMTS) in a buffer containing 100 mM HEPES (pH 7.4), 1 mM EDTA, and 2% SDS for 4 h at 40 °C. Blocked proteins were precipitated and then resuspended in buffer containing 100 mM HEPES (pH 7.4), 1 mM EDTA, and 1% SDS. Samples were incubated overnight with hydrated thiopropyl sepharose 6B resin and mixed with hydroxylamine (HA; pH 7.5) at a final concentration of 0.5 M. Negative control samples were incubated with Tris-HCl (pH 7.5) without HA. The supernatant (unbound fraction) was collected, and the resin was washed several times and then boiled in Laemmli buffer supplemented with 2.5% β-mercaptoethanol for 10 min. to elute the proteins (bound fraction). The volumes of unbound and bound fractions were equalized and subjected to SDS-PAGE followed by Western blotting.

### 4.5. Supplementation with Fatty Acids

Fatty acid supplementation of 2BT cells with either 100 µM Eicosapentaenoic acid {EPA; 20:5(n-3)} or Docosahexaenoic acid {DHA; 20:6(n-3)} was prepared in EMEM supplemented with 0.5% fatty acid-free BSA prepared as described below and added to the cells.

### 4.6. Lactate Dehydrogenase (LDH) Activity

LDH activity was measured as an indicator of cytotoxicity using the CyQUANT LDH Cytotoxicity Assay Kit (catalog #C20300; Thermofisher Scientific, Waltham, MA, USA) according to the manufacturer’s protocol. Briefly, 2BT cells were incubated with different concentrations of EPA and DHA (25, 50, 100 µM) for 15 h in quadruplicate. Following this, 50 μL of culture medium was transferred from each well into a new 96-well plate and mixed with 50 μL of the reaction mixture. The plate was incubated at room temperature for 30 min, protected from light, to allow enzymatic conversion of lactate to pyruvate, resulting in the formation of a red formazan product. The reaction was terminated by adding 50 µL of the stop solution. Absorbance was measured at 490 nm and 680 nm, and the LDH was calculated according to the manufacturer’s instructions.

### 4.7. Assessment of ASBT Function by [^3^H]-taurocholate ([^3^H]-TC) Uptake

[^3^H]-TC uptake was performed as previously described [14]. The cells were washed with uptake buffer containing 110 mM NaCl (with sodium) or choline chloride (without sodium), 4 mM KCl, 1 mM MgSO_4_, 1 mM CaCl_2_, 45 mM mannitol, 5 mM glucose, and 10 mM HEPES (pH 7.4), and then incubated with uptake buffer containing 1 μCi/mL of [^3^H]-TC and 10 μM cold taurocholate (TC) at room temperature for 5 min. The uptake was terminated by washing the cells with ice-cold PBS. The cells were then lysed in 0.5 M NaOH, and radioactivity was measured by liquid scintillation counting (Packard Tri-CARB 1600-TR, Packard Instruments, Downers Grove, IL, USA). Total protein was measured by the method of Bradford [39], and the results were calculated as pmol × mg protein^−1^ × 5 min^−1^. The Na^+^-dependent uptake was calculated by subtracting the values of the uptake in the absence of Na^+^ from those in the presence of Na^+^.

### 4.8. Assessment of ASBT Function by Bioluminescent Bile Acid Tracer (CA-SS-Luc) Uptake

Assessment of ASBT activity by CA-SS-Luc uptake was performed as recently described [40]. Cells were washed at room temperature with uptake buffer containing 110 mM NaCl (with sodium) or choline chloride (without sodium), 4 mM KCl, 1 mM MgSO_4_, 1 mM CaCl_2_, 45 mM mannitol, 5 mM glucose, 10 mM HEPES (pH 7.4), and 100 µM TC. Cells were then incubated with uptake buffer containing 1 μM CA-SS-Luc and immediately imaged on a Xenogen IVIS Spectrum in vivo imaging system (Caliper Life Sciences, Hopkinton, MA, USA); bioluminescence was captured continuously with 1–45-min exposures. The luminescence from each well was quantified using Living Image software; version 4.8.0 (Perkin Elmer, Shelton, CT, USA).

### 4.9. Metabolic Labeling with Alkyne Fatty Acids

Metabolic labeling was performed with 100 μM alkyne-PA (alk-PA) or 100 μM alkyne-EPA (alk-EPA) obtained from Cayman Chemicals, Ann Arbor, MI, USA. The procedure was modified from our previously published protocol [14]. Briefly, fatty acids were delivered into the cells in EMEM supplemented with 0.5% fatty acid-free BSA. To prepare these solutions, stocks of either unlabeled PA, alk-PA, or alk-EPA in DMSO were added to 20% fatty acid-free BSA. These solutions were briefly sonicated and incubated at 37 °C until the fatty acids had fully dissolved, then diluted 40× in 37 °C EMEM. These solutions were added to cells and incubated at 37 °C for 15 h to allow for the incorporation of fatty acids. Following incubation, cells were lysed in a buffer containing 150 mM NaCl, 50 mM HEPES, 2 mM MgCl_2_, 0.1% Triton X-100, 1% SDS, and 1× EDTA-free protease inhibitor. Up to 500 μg of protein lysate was subjected to copper-catalyzed azide–alkyne cycloaddition with azido-PEG3-biotin. Briefly, lysates were incubated for 1 h at room temperature with 250 μM azido-PEG3-biotin in a solution containing 1 mM CuSO_4_, 5 mM THPTA, and 15 mM sodium ascorbate. Proteins were precipitated in methanol, washed, and solubilized in the RIPA buffer. They were then incubated with NeutrAvidin agarose beads (Thermo Fisher) overnight at 4 °C with end-over-end mixing. The following day, supernatant (unbound fraction) was collected, the resin was washed several times with PBS, and bound proteins were eluted by boiling in Laemmli buffer supplemented with β-mercaptoethanol for 10 min. Samples were then subjected to SDS-PAGE followed by Western blotting.

### 4.10. Animals Studies

All the experimental procedures were approved by the animal care committee of Jesse Brown VA Medical Center (Chicago, IL, USA). 8–10-week-old mice (C57BL6/J) were obtained from Jackson Laboratories and acclimatized in the animal facility for 7 days. Mice were then fed for 12 weeks with a high-fat diet (HF) containing 42% kcal from fat and 0.5% cholesterol, or a high-fat diet supplemented with fish oil (HFFO), containing 6.5% menhaden oil as a source of n-3 PUFAs (Research Diets). The diet compositions are shown in Table 2. Mice had free access to food and water and were maintained in 12/12h light and dark cycle. At the end of the feeding period, the mice were humanely euthanized by CO_2_ anesthesia followed by cervical dislocation. The mice were weighed weekly throughout the entire protocol, and total weight gain was calculated for each individual mouse. Blood, intestine, and liver tissues were collected from all animals.

### 4.11. Hepatic Lipid Extraction and Analysis

Total lipids were extracted from the livers of mice as previously described [41]. Approximately 100 mg of liver tissue was homogenized in 1 mL of isopropanol by using a tissue grinder, followed by vigorous shaking for 45 min at room temperature. Lysates were centrifuged at 13,000 RPM for 10 min. The supernatant from each sample was divided into 2 equal parts: one for cholesterol measurement after adding Triton X-100, and the other for triglyceride measurement using the L-type Triglyceride M enzymatic kit from Wako Diagnostics, Mountain View, CA, USA.

### 4.12. Fecal Bile Acid Extraction and Total Bile Acid Assay

Fecal bile acids were extracted as previously described [42] with modifications. Briefly, fecal samples were collected from individually housed mice over a 48 h period, air-dried at room temperature for 1–2 days, weighed, and ground to a fine powder. An equal amount of fecal powder was used for bile acid extraction. Samples were treated with sodium borohydride, which reduces 3-keto-bile acids to 3-α-bile acids, followed by saponification with HCl and NaOH and overnight incubation at 120 °C. The extracts were filtered, evaporated under nitrogen gas and reconstituted in water. Bile acids were purified using C18 solid-phase extraction columns preconditioned with methanol and water, washed with 20% methanol, and eluted with 100% methanol. Total bile acid concentration was measured using the Mouse Total Bile Acid Assay Kit (Catalog: 80471) from Crystal Chem (Itasca, IL, USA) according to the manufacturer’s instructions.

### 4.13. Histopathological Analysis

For Oil Red O staining, frozen sections from the mouse liver were cut at 10 µm. Sections were air-dried, fixed in formalin for 10 min, washed with running tap water for 10 min, and then rinsed with 60% isopropanol. Sections were then incubated with a freshly prepared working solution of Oil Red-O (Fisher Scientific) for 15 min and then rinsed with 60% isopropanol. Nuclei were stained with alum hematoxylin and rinsed with water. Slides were mounted in glycerol, and staining was assessed under the microscope.

### 4.14. Statistical Analysis

Results were expressed as mean ± SEM of at least three experiments performed on multiple occasions. For the animal studies, each data point in the figure represents a single measurement from an individual mouse, and results were expressed as the mean ± SD unless otherwise indicated. Statistical analysis was performed by Student’s *t*-test (GraphPad Prism; version 11.1.0). *p* ≤ 0.05 was considered statistically significant.

## 5. Conclusions

The results of the current studies provide a novel platform that should prompt future research to address important remaining questions. For example, future studies will need to assess the molecular basis of EPA binding to ASBT. Also, developing an in vivo bioluminescence-based approach will be essential to directly measure, in real time and in live animals, the dynamics of bile acid absorption during fish oil feeding and diets enriched with other types of fatty acids. Overall, the data presented in this study reveal novel regulatory pathways that modulate bile acid absorption and may be targeted as effective adjunct therapies to enhance the treatment of bile acid-related disorders.

## Figures and Tables

**Table 1:** PCR primers.

Mouse Genes		
Gene	Forward	Reverse
**zDHHC1**	ATGAACATCTGCAACAAACCCT	GCTCCATCCATTCCTTCGAGAG
**zDHHC2**	CTACTACGCCTACGCCATCC	TCCAGCAATTCTTTCTCTGCAT
**zDHHC3**	ATCCCCACCCATCACTTCC	CTCGGATAAACCACATGGTC
**zDHHC4**	TGATTTGTGTTGTCCTGATCTGC	GGAGGCACTGCGGGATTAC
**zDHHC5**	AAACCCAGCAAGTATGTACCG	AATTGCATTGTAAATGGGCACTG
**zDHHC6**	GAGTAAGAGGGTGGTTTCCTAGA	GCTGGATCTGAGTCACCATCAC
**zDHHC7**	CGGGACATCGAGCACCATC	CGAAGTCTGCATAGACGACGA
**zDHHC8**	CCCTCTCCTGTGCGCTATG	TCGCCAAAGAGTGAGTCAGTC
**zDHHC9/10**	AAGGTGACACGGAAATGGGAG	CGACACTCGAAGGCAAAGAA
**zDHHC11**	CTGACACCAATGTCCGACTCA	CGCGGTAACCTCACACAGG
**zDHHC12**	CCCTGCTGCTTTATCTGGCT	TGCGTTCACCCACACAGTTC
**zDHHC13**	TCGCAGTGCAGGAATCACAG	GGCAGCCCAGTGAAGAAGA
**zDHHC14**	CACACTCTCAGACATTATGCCC	TGCATGGTACGGCTATGTGC
**zDHHC15**	GGTGCCAGTGCTCGTTATTGT	AACTTTTTCCGCTGGACTCAA
**zDHHC16**	CAGCGAAGTCTGCTGTTGG	CAGCGGATCACATTGTCCAC
**zDHHC17**	AGACGATAGTTGAACTTGCAGAG	ACCTACACAGTTACCCACCCA
**zDHHC18**	TCAACGGGCAGACAGTGAAAC	GAAGCGGTAGTTCCGTCTCC
**zDHHC19**	TGTGACACTTGTGAAGGAACC	AAAAACAGCAGCAGCGTTACA
**zDHHC20**	GGAAAGACCGTTGTTTACCTTGT	ACTCCTTCTCATAACGCTCCTTC
**zDHHC21**	ATGGGTCTTCGGATTCACTTTG	CCCTCACTAAGGCAACCAGG
**zDHHC22**	CGGCTGCTCAACGTGGTAG	CCAGGACGTAATTGCCCAGG
**zDHHC23**	CAACAGCCAAATTGAATGCCC	AACATCGTCCTTCAGTGTGCG
**zDHHC24**	GCTTACTGTTACCAGTGCCAA	ACACAGCAGCCCAATAGGC
**zDHHC25**	TCCTCGTGGGTCCAAAACC	GCACAGGATGCCAATGGGA
**ASBT**	TGGGTTTCTTCCTGGCTAGACT	TGTTCTGCATTCCAGTTTCCAA
**SHP**	GCACGATCCTCTTCAACCCA	CAGAAGGGTGCCTGGAATG
**CYP7A1**	AGCAACTAAACAACCTGCCAGTACTA	GTCCGGATATTCAAGGATGCA
**CYP27A1**	CCAGGCACAGGAGAGTACG	GGGCAAGTGCAGCACATAG
**CYP8B1**	GACCACAGGATGATCCACTTAGC	ACCTTTAGGCCCTAGCATCAC
**GAPDH**	TGTGTCCGTCGTGGATCTGA	CCTGCTTCACCACCTTCTTGAT
**Human genes**		
**zDHHC1**	CAACTTGTGCAACGTGGATGT	AAGAGCCGGTAGTTCCGCT
**zDHHC2**	TCCCGGTGGTGTTCATCAC	CAACTTGTTCGCCAGTGTTTTC
**zDHHC3**	CCACTTCCGAAACATTGAGCG	CCACAGCCGTCACGGATAAA
**zDHHC4**	TGATGGGTCTTGTTCTTATCTGC	AGGTGTGGTTTCTCGTATGGAA
**zDHHC5**	CACCTGCCGCTTTTACCGT	CGGCGACCAATACAGTTATTCAC
**zDHHC6**	AGTCTGCCAAGCATACAAGGC	CCAGTGGTGCTAAAAGGAGAAAC
**zDHHC7**	CTGACCGGGTCTGGTTCATC	CATGACGAAAGTCACCACGAA
**zDHHC8**	CTGTTCCCGTCTACAATGGCA	GACCTGGATACCTCGCACAT
**zDHHC9**	CCTGGGTGGGGAATTGTGTT	ACGACGGACCAGAGTGTAAAG
**zDHHC11**	AGTGAACGGCTGGTCGTTAC	CCACGTAGGCAATGTATTTCCA
**zDHHC12**	GTGCTGACCTGGGGAATCAC	CTGCACATTCACGTAGCCA
**zDHHC13**	ACCCCACTCTTATTGATGGAGA	TGTCTGCCCATTTACATCTGTC
**zDHHC14**	TGTGATAACTGCGTAGAACGGT	CGTGGGTGATAACGAATGCAA
**zDHHC15**	GGTGCCAGTGCTCGTTATTGT	AAGACGTAGGCATAGTAGGACC
**zDHHC16**	ACTCCGGGGTCTAGTACAGC	CCAGCGGATCACGTTGTCT
**zDHHC17**	GGCCCGGATGAGTACGATAC	TCCAAGAGGTTCACCATACCA
**zDHHC18**	CACCCCGAACCTCACACTG	TGAAGGCCGTCAGGAATGAGA
**zDHHC19**	TTGCTGCCTTCAATGTGGTG	CGGAGCCTTGATGTAAGATGC
**zDHHC20**	CGCACCCACGTTTTCATACG	TCTGGCATACTCATTCTGGTTTG
**zDHHC21**	TGTTGTTGACCCACATGGTTG	GAGGCCCTCACTAAGGCAA
**zDHHC22**	GGGGCGCTCTTCCTATTCC	GCAGAAGTGGGTGCTAGGTG
**zDHHC23**	TCTGGATGAAGGGTGTGATCG	GCTCCCCTAAGCCAAGGAA
**zDHHC24**	CTGGCACAGTTTGCCTTGG	CAGGACCCAGGTCATAGGAG
**GAPDH**	GAAATCCCATCACCATCTTCC	AAATGAGCCCCAGCCTTCT

**Table 2:** Diet composition.

	D19112105		D19112106	
	High Fat/Milk Fat/0.5% Chol		High Fat/Milk Fat/Fish Oil/0.5% Chol.	
	gm%	kcal%	gm%	kcal%
Protein	23.1	20	23.1	20
Carbohydrate	44	38	44	38
Fat	21.6	42	21.6	42
Total		100		100
kcal/gm	4.63		4.63	
Ingredient	gm	kcal	gm	kcal
Casein, 80 Mesh	200	800	200	800
L-Cystine	3	12	3	12
Corn Starch	276	1104	276	1104
Maltodextrin 10	100	400	100	400
Cellulose, BW200	50	0	50	0
Corn Oil	10	90	10	90
Menhaden Oil, 200 ppm tBHQ	0	0	56	504
Milk Fat, Anhydrous	135	1215	79	711
Palm Oil	44	396	44	396
tBHQ	0.02	0	0.009	0
S10026	10	0	10	0
DiCalcium Phosphate	13	0	13	0
Calcium Carbonate	5.5	0	5.5	0
Potassium Citrate, 1 H_2_O	16.5	0	16.5	0
Vitaminutes Mix V10001	10	40	10	40
Choline Bitartrate	0	0	0	0
Cholesterol	3.85	0	3.85	0
FD&C Yellow Dye #5	0	0	0	0
FD&C Red Dye #40	0.05	0	0	0
FD&C Blue Dye #1	0	0	0.05	0
Total	876.92	4057	876.909	4057
Palmitic Acid (g%)	6.27		5.54	
Menhaden Oil (g%)	0		6.39	
Menhaden Oil (kcal%)	0		12	
tBHQ (mg/4057 kcals)	20		20	
Cholesterol (%)	0.49		0.51	

## Data Availability

No new data were created or analyzed in this study. All data are fully integrated within the main manuscript.

## Supplementary Materials

The following supporting information can be downloaded at: https://www.mdpi.com/article/10.3390/ijms27219920/s1.
